# Growth Kinetics of the Selected Intermetallic Phases in Ni/Al/Ni System with Various Nickel Substrate Microstructure

**DOI:** 10.3390/nano9020134

**Published:** 2019-01-22

**Authors:** Izabella Kwiecien, Piotr Bobrowski, Anna Wierzbicka-Miernik, Lidia Litynska-Dobrzynska, Joanna Wojewoda-Budka

**Affiliations:** Institute of Metallurgy and Materials Science, Polish Academy of Sciences, 30-059 Krakow, Poland; p.bobrowski@imim.pl (P.B.); a.wierzbicka@imim.pl (A.W.-M.); l.litynska@imim.pl (L.L.-D.); j.wojewoda@imim.pl (J.W.-B.)

**Keywords:** intermetallic phases, growth kinetics, Al–Ni system

## Abstract

Reactivity in nickel–aluminum system was examined for two variants of nickel substrates in terms of the size and shape of Ni grains. The microstructure transformation aroused due to the annealing at 720 °C for different annealing times (0.25 to 72 h) was consequently followed. The sequence of formation of the particular intermetallic phases was given. The interconnection zones were examined by means of scanning electron microscopy supported with energy dispersive X-ray spectroscopy and electron backscattered diffraction techniques as well as by the transmission electron microscopy. The growth kinetics data for AlNi, AlNi_Ni-rich_ and AlNi_3_ phases for both variants of substrates was given, indicating the differences obtained in previous works on this subject.

## 1. Introduction

Nickel–aluminum system is well known and commonly researched reactive system. This is associated with the wide practical application of the AlNi_3_ and AlNi intermetallic phases, which are used on a large scale in many industrial branches for example, as multilayers, oxidation resistant coatings (aluminizing treatment of nickel alloys), turbine blades (aircraft industry), electronics industry. To ensure the protection against the oxidation, aluminum-rich phases such as Al_3_Ni, Al_3_Ni_2_ and AlNi are the most important ones, however, the greatest mechanical properties are associated with the presence of only two phases: AlNi and AlNi_3_ [1,2,3].

β-AlNi and γ-AlNi_3_ are cubic phases possessing B2 and L12 structure types, respectively. These intermetallics are characterized by high degree of order at elevated temperature and indicate high mechanical strength even at high temperature. Also they possess high thermodynamic stability in wide range of chemical composition and high degree of crystal lattice order with various amounts of defects. β-AlNi phase composition varies in the wide range from 40 to 55 at. % of Al at 700 °C. Additionally, within its structure many defects can be formed. Bradley and Taylor [4] have shown that nickel replaces aluminum in its lattice causing the excess of the nickel, however, in Ni-deficient compounds, aluminum does not replace the nickel in its lattice, causing formation of vacancies. It explains high number of structure’s defects. As a result of high temperature diffusion processes, γ-AlNi_3_ intermetallic phase can be created in the reaction between AlNi and Ni. Base structural Ni_3_Al cell crystallizes in cubic lattice, where aluminum atoms are in the corners and nickel atoms are at the center of cell walls [4,5,6].

Many works consider the diffusion processes in Ni-Al system, however, as variables, only parameters such as annealing time and temperature of reaction either in solid state or solid/liquid state are analyzed. Additionally, the system is studied in many configurations with respect to the chemical composition of initial substrates (end members) and various their combinations [3,5,7,8,9,10,11]. Moreover, different experimental procedures have been employed in these studies such as: Ni/Al diffusion couples [3,5,8,9,10,12], Ni/Al/Ni sandwiches [7,13,14,15], or more complex assemblies such as multilayers, nanocoatings or aluminized nickel alloys [16,17,18,19,20,21].

**Figure 1:**
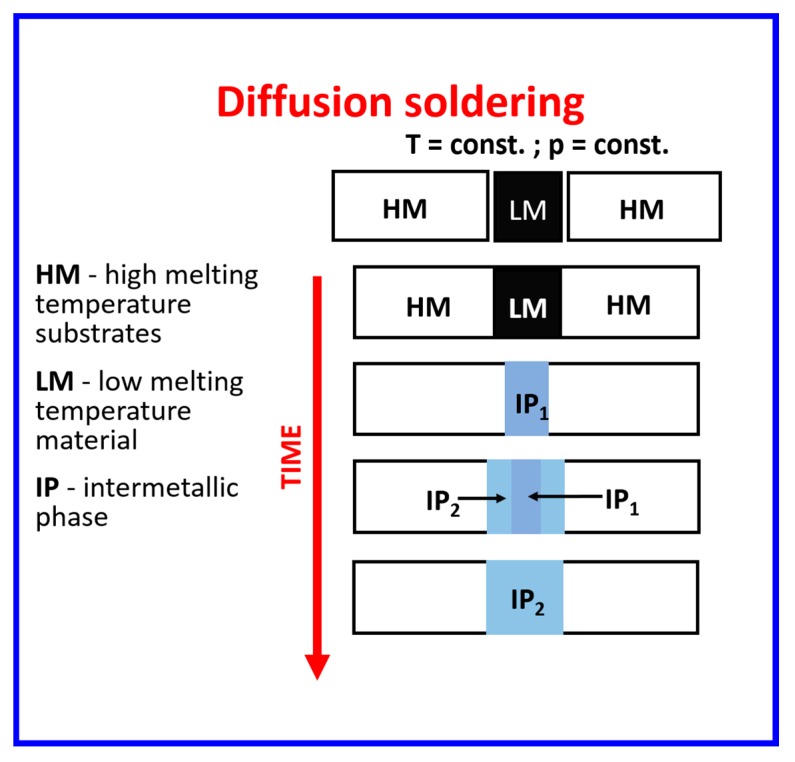
Schematic of diffusion soldering process [22].

One of the promising joining processes is the diffusion soldering (DS) [13,22,23,24], which is schematically presented in Figure 1. The main stage of this process is the isothermal solidification, where the reaction of liquid low melting interlayer (LM) and solid substrates (HM) takes place. With increasing of the DS temperature to the appropriate one—required in the process, the low melting component turns into the liquid state and the reaction at the solid/liquid interface (between the high and low melting components) begins. After some time, the first intermetallic phase is formed, then, in the next stage of DS diffusion in solid state between the obtained intermetallic phase and the high-melting substrate takes place. When the whole liquid metal is consumed, reaction proceeds only in the solid state. Phases are created and consumed one by one or simultaneously due to inter-diffusion, being replaced by the phases enriched more and more in element of higher melting point.

**Figure 2:**
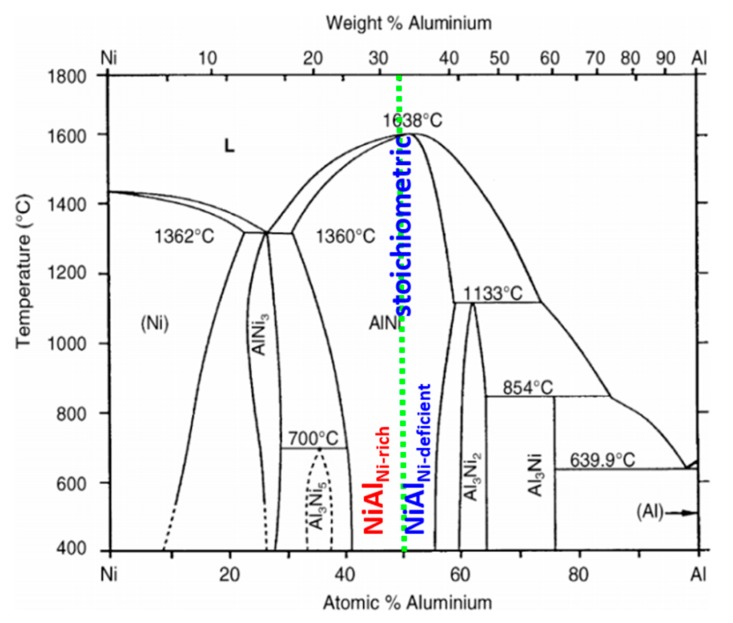
Equilibrium phases diagram Ni–Al with distinction between various types of the AlNi phase [25].

In the Ni/Al/Ni system the sequence of appearance of the intermetallics can be predicted based on the Al-Ni equilibrium phase diagram (Figure 2) [25]. The phases are being created going from the lower melting to higher melting component. What is interesting, there are contradictory literature reports concerning the sequence of the intermetallics formation in Ni/Al system, regarding the order of formation of the high aluminum intermetallic phases such as Al_3_Ni and Al_3_Ni_2_. Two opposite approaches are possible. First one involves situation, when as a primary phase Al_3_Ni phase precipitates and then Al_3_Ni_2_ one is created [13,14,26]. Some modeling results and in-situ experiments indicated on formation of Al_3_Ni_2_ phase as the first one and then Al_3_Ni [27,28,29]. As the process proceeds, the intermetallic compounds such as: AlNi, Al_3_Ni_5_ and AlNi_3_ are formed [13]. In the cited works the chemical composition was verified mainly by EDS technique in SEM [13,14,26,27,28,29]. Additionally, in [26,29] works, the XRD measurements were also conducted to confirm the phases’ composition present in the interconnection zones, while in [27,28] for the phase sequence occurrence the mathematical model was proposed.

Literature’s survey also presents the validity of diffusion studies on Ni substrates of variable grain boundaries. Works of Yu-Chen Tseng et al. [30] based on liquid–solid Sn/Ni system indicated significant differences in the course of the diffusion process conducted with applied commercial coarse-grained Ni substrates and fine-grained Ni layer electroplated (e-Ni) on Ba_2_Te_3_. In both type of samples after the annealing, the Ni_3_Sn_4_ phase is formed, but for e-Ni substrate, additional Ni_3_Sn_2_ phase in the form of the thin film between Ni and Ni_3_Sn_4_ appeared. A large number of grain boundaries in e-Ni layer, being fast diffusion channels for Ni atoms towards Ni_3_Sn_4_, resulted in the growth of this additional phase, consisting of more nickel. The additional layer suppressed the growth of Ni_3_Sn_4_ on e-Ni layer, which is thinner in comparison to the one, formed using coarse grained Ni substrates. A similar experiment in solid Ni/liquid Al/solid Ni system is more difficult to analyze, as higher joining temperature is necessary to apply than the one for Sn/Ni diffusion pair and the nickel recovery and recrystallization phenomena occurs.

This paper shows the results concerning the relation between the reactivity (intermetallic phases growth kinetics) in Ni/Al system and the microstructure of nickel substrates. As the grain boundaries can act as fast diffusion paths, they can influence the growth kinetics of the intermetallic phases and therefore, different results can be reported for apparently the same experimental procedure. In this work Ni substrates of two crystallographic orientations was employed to study the growth of the intermetallic phases in solid/liquid and solid/solid state in Ni/Al/Ni system.

## 2. Materials and Methods

**Figure 3:**
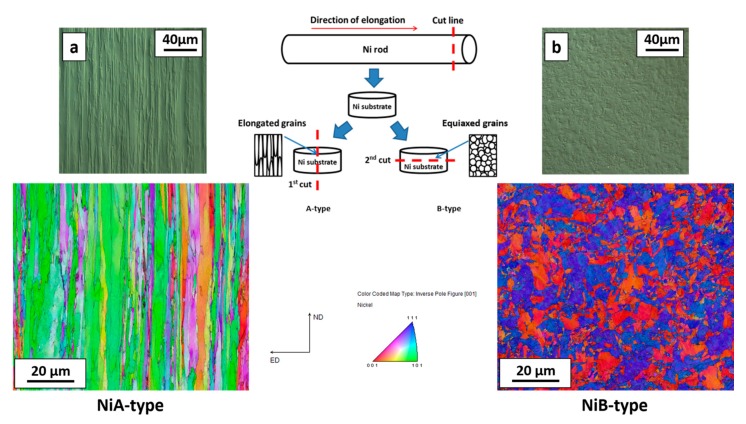
The scheme of the preparation of nickel substrates with their optical microstructures and electron backscattered diffraction (EBSD) maps (**a**) NiA-type, (**b**) NiB-type.

Substrates used in the experiment were prepared from high purity commercial Ni rod (99.999, (Goodfellow Cambridge Ltd., Huntingdon, UK) with a diameter of 5 mm. The rod was cut in two orientations: along and perpendicular to the rod elongation direction as it is showed in Figure 3. EBSD maps revealed the different crystallographic orientation of both types of substrates. Optical microstructures and EBSD maps show significant difference in the appearance of both substrates, for substrate NiA-type (Figure 3a) grains are elongated and narrow. On the other hand, for the NiB-type substrate (Figure 3b), grain possess irregular shape and on the map considerable refinement of the structure is visible.

In order to obtain the diffusion-soldered interconnections, two nickel slices with the same or different orientation were grinded, polished and cleaned in ultrasonic cleaner for 300 s. Then the thin (80 µm) slice of high purity aluminum (99.999, Goodfellow Cambridge Ltd., Huntingdon, UK) was clamped between two Ni substrates and held in specific temperature for different periods of time. As the aluminum melting point is of 660 °C, the 720 °C was applied as the joining temperature. Such temperature, higher than the one necessary to melt Al, was chosen for several reasons. First of all, it ensures that Al passes to the liquid state during the annealing in the vacuum. It also allows comparing obtained results with the data presented by Lopez et al. [13]. Only slight mechanical pressure was used to avoid of leakage of the solder and the samples were sealed in quartz ampules to prevent the samples’ oxidation. Table 1 shows variety of applied experimental assemblies and conditions of their annealing.

The cross-sections of the interconnections for scanning electron microscopy (SEM, Quanta 3D FEG, FEI, Hillsboro, OR, USA) examinations were prepared by standard metallographic procedure: embedding samples in epoxy, grinding and then polishing with the diamond paste (3 μm) and silica (0.04 μm). As a starting point the scanning electron microscopy observations with 20 kV accelerating voltage and energy dispersive X-ray spectroscopy (EDS, Trident (EDS-EBSD-WDS), EDAX Inc., Tilburg, The Netherlands) analysis were carried out for each sample, revealing its phase composition and thickness of particular intermetallics layers and also the chemical composition changes across them. The samples in SEM were inspected using the backscattered electrons mode (BSE). As a next step, the electron backscattered diffraction technique was used to expose the amount and character of the grain boundaries and to correlate it with the creating phases composition and thickness. The thin foils for the transmission electron microscopy (TEM, TECNAI G2 200 kV, FEI, Hillsboro, OR, USA) observations were prepared using the Focused Ion Beam (FIB, Quanta 2D, FEI, Hillsboro, OR, USA) technique. It is the only technique suitable for this type of thin foils (exact location of the place of interest). During the milling process, problems with uneven consumption of the sample material were encountered. Difficulties were associated with significant difference in hardness of the various intermetallics phases located at the reaction zone. The obtained thin foils with the thickness of about 100 nm were next examined by TEM.

## 3. Results and Discussion

### 3.1. Sequence of Intermetallic Phases in Interconnections

The diffusion-soldering at the temperature of 720 °C for different periods of time resulted in the growth of the several intermetallic phases in the joined area. It is important that the sequence of phase-creation in all cases was the same as it was predicted in [13,14,26]. They grew according to equilibrium phases diagram from the ones rich in low melting component to the ones with higher amount of nickel. Sequence of their appearance in the interconnection zone depended on the duration of reaction.

**Figure 4:**
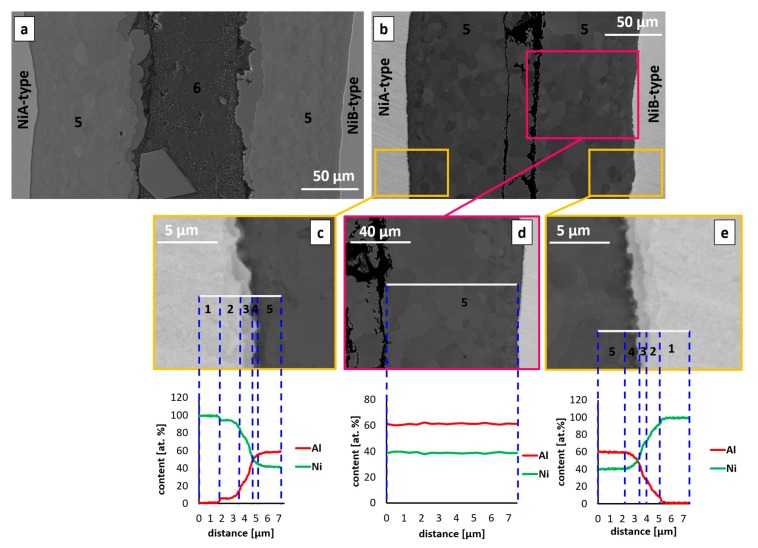
The SEM microstructures after (**a**) 15 min and (**b**) 30 min of reaction at 720 °C. The SEM microstructures in (**c**–**e**) present the magnified area of Al_3_Ni_2_/Ni interface. Numbers 1–6 denote various phases: 1-Ni, 2-Ni solid solution, 3-AlNi_3_, 4-AlNi, 5-Al_3_Ni_2_, 6-(Al)+Al_3_Ni.

The initial stage of reaction in Ni/Al/Ni interconnection was observed after 15 min of annealing (Figure 4a). SEM observations using BSE mode showed the contrast differences at the Ni/solder interface, pointing the existence of two intermetallic phases. The measurements of the chemical composition within the interconnection zone confirmed that these phases were Al_3_Ni (76.0 at. % Al, 24.0 at. % of Ni) and Al_3_Ni_2_ (60.6 at. % of Al, 39.4 at. % of Ni). On the other hand, the middle of the joined zone was composed of Al_3_Ni-Al eutectics (97.1 at. % of Al, 2.9 at. % of Ni) instead of pure aluminum. Moreover, inside of the Al_3_Ni-Al eutectics, the primary precipitates of the Al_3_Ni intermetallic phase possessing the faced walls could be observed. Thanks to the channeling contrast, the dual-morphology of Al_3_Ni_2_ is visible, showing the larger grains to be located closer to the middle of the interconnection and finer grains being located close to the nickel substrates. As it is showed in Figure 4a, the Al_3_Ni phase, growing next to the nickel substrate, formed the areas of irregular shape at the interface with eutectics. Such a morphology is called scallops and it is typical for the growth of the intermetallics with assistance of the liquid. Additionally, it was noticed that the interface between Al_3_Ni and Al_3_Ni_2_ phases is wavy. At this point it can be summarized that the interconnection consisted of the following constituents:

**Ni/Al_3_Ni_2_/Al_3_Ni/[(Al)+Al_3_Ni]_eutectics_/Al_3_Ni/Al_3_Ni_2_/Ni.**

The interface between nickel and Al_3_Ni_2_ phase is smooth and approximately parallel to the surface of the applied substrates. This type of planar interfaces is characteristic for solid/solid reaction. Similar observation was noticed in other papers [14,26].

The obtained interconnection zone after 15 min of annealing at 720 °C may be compared with joint described by Tumminello and Sommadossi [14], for the same time of annealing (15 min) but at higher temperature of 776 °C. The sequence of the intermetallics creation is in both cases compatible. In present work, the interconnection zones are much broader in comparison to the ones shown in [14], being approximately twice as wide. Table 2 contains the comparison of the results from both papers. In present work the whole joint consists of Al_3_Ni in 21% and Al_3_Ni_2_ in 59%, the rest is the eutectics (Al) + Al_3_Ni. In Tumminello’s work [14] the amount of the phases were 13% and 47%, respectively. The observed differences between these two works could arise mostly from the various thickness of applied aluminum foil (much broader in [14]) and also possibly from the used atmosphere (here vacuum, while argon in [14]).

Elongation of the reaction time to 30 min, allowed observing significant difference of the interconnection zones in comparison to the sample after 15 min of annealing. Figure 4b shows the interconnection at this stage of annealing, where the middle of the joint is completely filled by Al_3_Ni_2_ phase, which was confirmed by EDS. The line scan through the area inside the pink frame, allowed excluding the presence of the eutectics (Al_3_Ni–Al) and Al_3_Ni phase in this area. The next important microstructural feature exists at the interface between Al_3_Ni_2_ phase and Ni substrate and has occurred in early stages of solid/solid reaction. The EDS measurements indicate high variability of chemical composition in the areas marked with yellow frames. Average content of aluminum in Al_3_Ni_2_ equals 60.5 at. %, while in the close neighborhood of the Al_3_Ni_2_/Ni interface it is about 57.6 at % and decreases in the direction toward the nickel substrate. The next measurements of the chemical composition showed the presence of narrow zones of about 1.5 µm, where the content of aluminum equals 51.3 at. %, which can be attributed to AlNi phase, then 30.3 at. % of Al, standing for AlNi_3_ phase. The presence of Ni solid solution was also observed (0.8 at. % of Al). In the mentioned area the differences in BSE contrast can be observed, the last one being distinguishable at the Al_3_Ni_2_/AlNi interface. Changes of the chemical composition near the phases’ boundaries indicate progressive diffusion processes leading to the initial stages of the AlNi growth after 30 min of annealing. Interconnection zone constituents can be this time summarized as follows:

**Ni/Ni_solidsolution_/AlNi_3_/AlNi/Al_3_Ni_2_/AlNi/AlNi_3_/Ni_solidsolution_/Ni.**

The interfaces of the phases growing due to the reaction in the solid state show a linear character, as it was the case in the samples being annealed for 15 min shown in Figure 4a.

**Figure 5:**
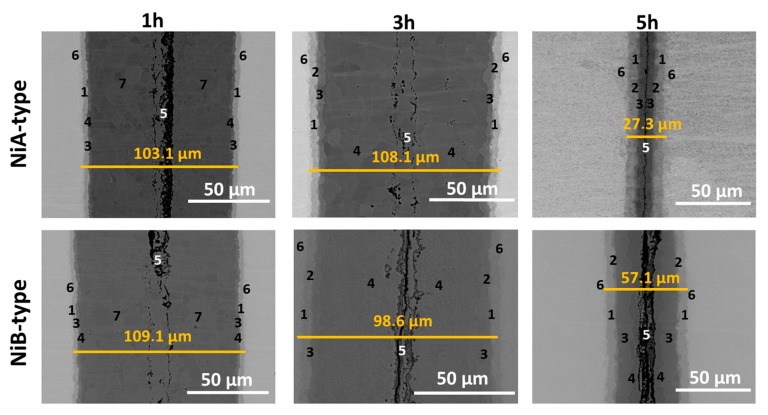
Microstructure of the Ni/Al/Ni couples obtained at 720 °C after annealing for: 1, 3, 5 h using A- and B- type of Ni substrates. Numbers 1–6 denote particular intermetallic phases: 1-AlNi_3_, 2-AlNi_Ni-rich_, 3-AlNi, 4-AlNi_Ni-deficient_, 5-(Al) + Al_3_Ni, 6-Ni solid solution, 7-Al_3_Ni_2_.

Figure 5 shows the morphology of the growing phases after 1, 3 and 5 h of reaction at 720 °C. SEM observations revealed the existence of several intermetallic phases. The measurements of the chemical composition within the joined area indicated that they consisted of: 

After 1 h:

**Ni/Ni_solidsolution_/AlNi_3_/AlNi/AlNi_Ni-deficient_/Al_3_Ni/[(Al)+Al_3_Ni]_eutectics_/Al_3_Ni_2_/AlNi_Ni-deficient_/AlNi/AlNi_3_/Ni_solidsolution_/Ni **

After 3 h:

**Ni/Ni_solidsolution_/AlNi_3_/AlNi_Ni-rich_/AlNi/AlNi_Ni-deficient_[(Al)+Al_3_Ni]_eutectics_/AlNi_Ni-deficient_/AlNi/AlNi_Ni-rich_/AlNi_3_/Ni_solidsolution_/Ni **

**Figure 6:**
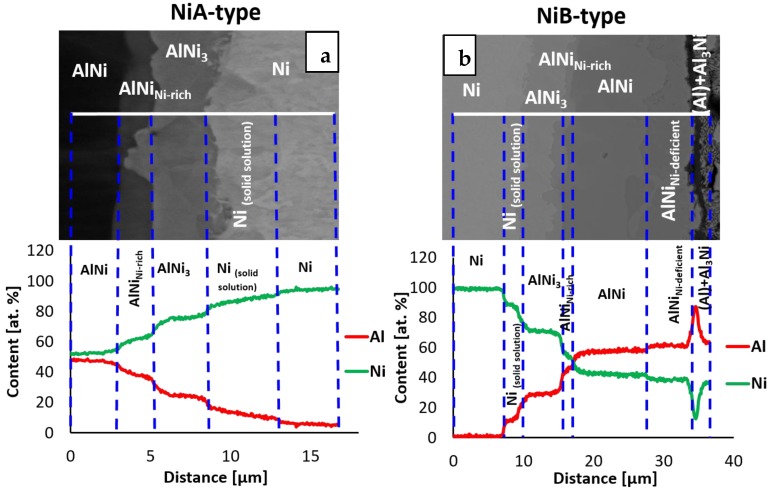
SEM micrographs of the cross-sectional view of a Ni/Al/Ni interconnections after 5 h of reaction time at 720 °C and EDS line-scan across this area for substrates of A-type (**a**) and B-type (**b**).

Obtained results are the same for both types of applied orientations of nickel substrates. However, after 5 h of reaction time one significant difference was noticed. Namely, the sequence of the intermetallic phases for types A and B was not the same (Figure 6). For Ni/Al/Ni interconnection, where the substrates of type B were used, the phase sequence was the same as in the case of 3 h of annealing, whereas, for the joints obtained from substrates of A-type, the AlNi phase deficient in nickel (AlNi_Ni-deficient_) did not appear. Figure 5 presents the phase composition of the interconnection zones for both types of substrates, where particular phases (as previously) are noted by numbers. As can be seen in the equilibrium phase diagram presented in Figure 2, the AlNi phase possesses a wide range of chemical composition. Therefore, there are several types of AlNi intermetallics, namely: stoichiometric, where the ratio of Ni to Al equals (50:50 at. %), AlNi deficient in nickel (45–50 at. % of Ni) and AlNi rich in nickel (50–60 at. % of Ni). Last one was evidenced in the literature data by Lopez et al. [13].

**Figure 7:**
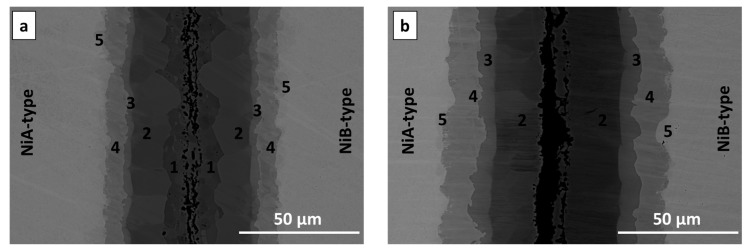
SEM microstructures of the Ni/Al/Ni couples obtained at 720 °C after annealing for: (**a**) 20 and (**b**) 72 h using A- and B- type of substrates of Ni. Numbers 1–5 denote particular intermetallic phases: 1-AlNi_Ni-deficient_, 2-AlNi, 3-AlNi_Ni-rich_, 4-AlNi_3_, 5-Ni solid solution.

A significant expansion of the annealing time to 20 and 72 h resulted in widening (Figure 7a) and disappearance (Figure 7b) of particular phases. After 20 h annealing at 720 °C the phases are broadened, however, the location of phases within the interconnection is the same as for samples annealed for 5 h using the substrates of B-type. The interconnection zone is symmetric. The AlNi deficient in nickel is slowly consumed, while the stoichiometric phase expands. 72 h is enough time to fully consume AlNi deficient in nickel and in the interconnection area only AlNi (51.6 at. % Ni), AlNi rich in nickel (60 at. % Ni) and Al_3_Ni (24.1 at. % Ni) are present. Phases of AlNi type are approximately twice wider in comparison to 20 h of annealing, AlNi_3_ phase changes its thickness of about 30% (broadening). The sequence of the phases after 72h is as follow:

**Ni/Ni_solidsolution_/AlNi_3_/AlNi_Ni-rich_/AlNi/AlNi_Ni-rich_/AlNi_3_/Ni_solidsolution_/Ni. **

Average chemical composition of all intermetallic phases after annealing for different periods of time is collected in Table 3.

**Figure 8:**
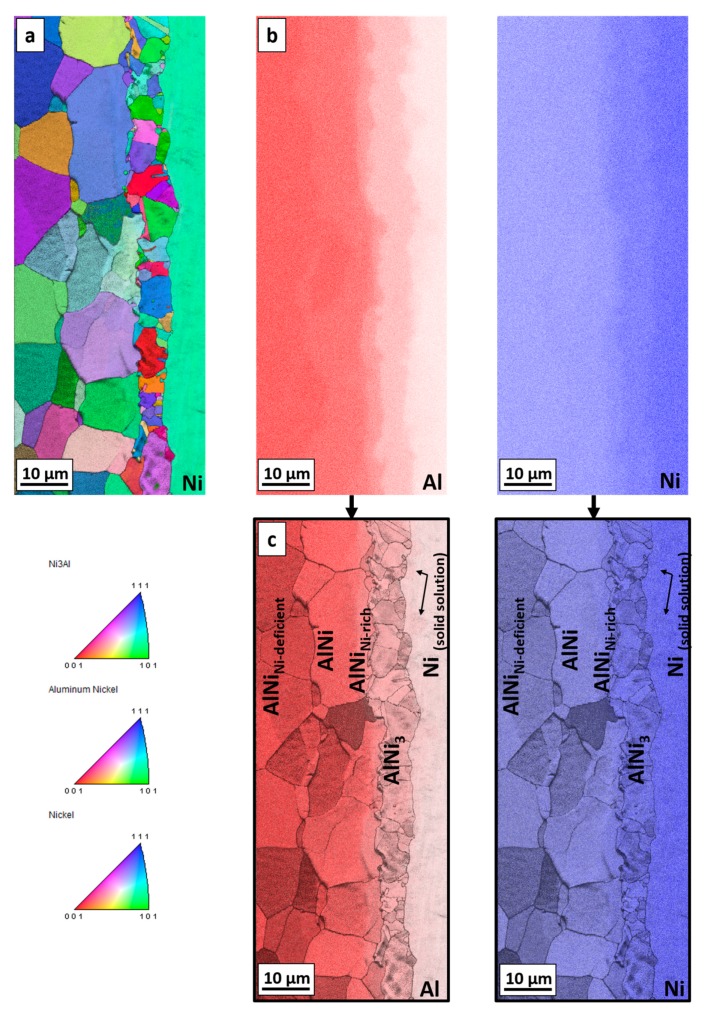
(**a**) EBSD map with (**b**) EDS maps of Al and Ni elements distribution for the sample annealed for 20 h at 720 °C. (**c**) The imposition of the EBSD indexing confidence map and EDS map analysis for Al and Ni elements.

**Figure 9:**
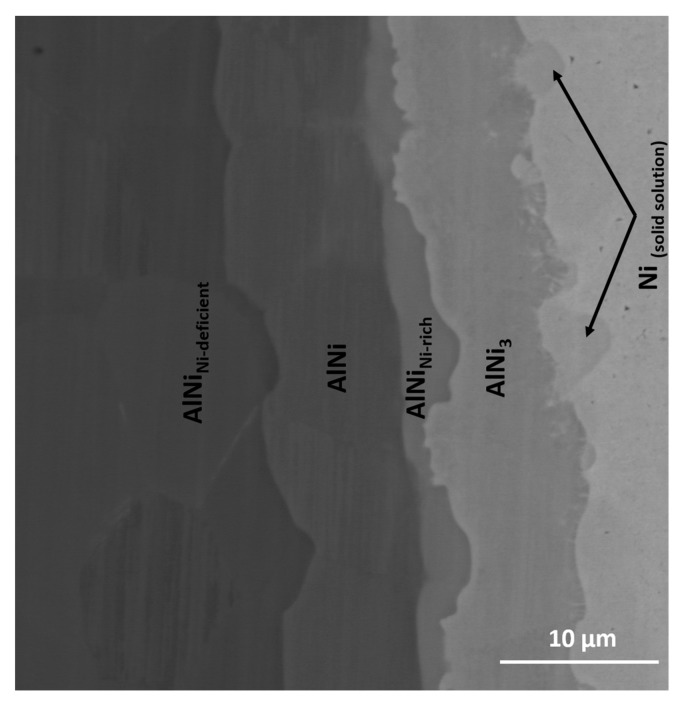
SEM micrograph in BSE mode of the intermetallic phases formed in the solid state after 20 h at 720 °C.

The interfaces, where the solid/solid diffusion occurs were also examined by EBSD technique. Due to sufficient width of particular phases, which grow with time, the sample annealed for 20 h at 720 °C was selected. The EBSD map in Figure 8a indicates the existence of three main areas, which are indexed starting from the nickel as: Ni, AlNi_3_ and AlNi. The map shows that the zone of interest consists of grains with a random crystallographic orientation and the one large grain of nickel. The differences in size of grains for particular areas are visible. Much finer grains in comparison to other areas are observed for the intermetallic phase identified as AlNi_3_. On the other hand, in the case of AlNi phases large grains are observed. In both cases range of these phases grain sizes is variable. Complementary to the EBSD map, for the same area the EDS maps were collected (Figure 8b). As can be noticed, these EDS maps show more individual phases compared to EBSD measurement. Area between Al_3_Ni and AlNi intermetallic phases is rich in nickel, in comparison to AlNi phase, while at the second side of AlNi phase, the area deficient in nickel is present. This dependency is compatible with SEM micrograph registered in BSE mode for the same area and it is shown in Figure 9. For full understanding of the observed relation, the EBSD indexing confidence map was imposed in the EDS maps for Al and Ni elements (Figure 8c). The result was surprising, as it was mentioned above, the EBSD map does not indicate the existence of more than three phases. However, the combination of EBSD and EDS maps reveals the concentration gradient throughout the AlNi phase grains. Some grains of AlNi phase are enriched in nickel, confirming previous suspicion of existence of AlNi rich phase. The most interesting observation is that the AlNi Ni-rich phase does not create new grains but rather changes the composition of the grains of already existing phases. In contrary to this, AlNi deficient in nickel possess own grains, being separated from the AlNi stoichiometric phase ones.

**Figure 10:**
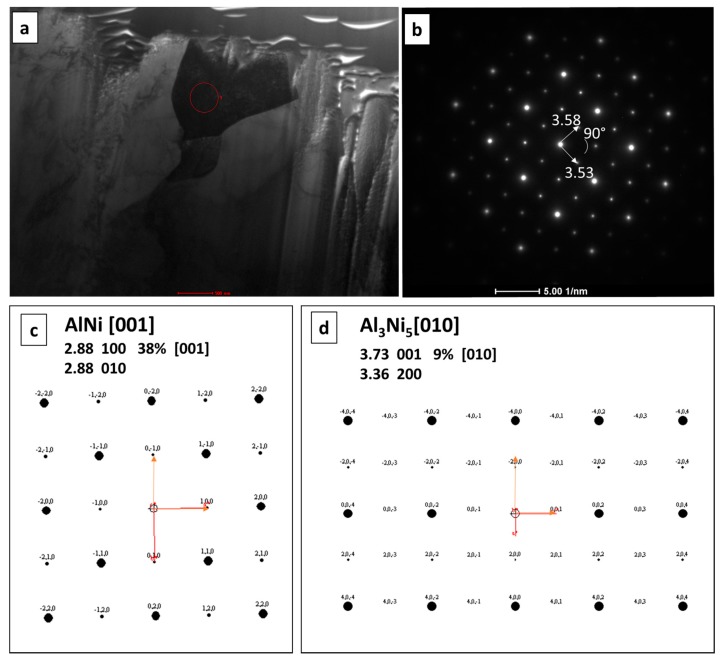
(**a**) TEM bright field image showing the microstructure of the AlNi intermetallic phase for sample annealled for 3 h at 720 °C together with the corresponding (**b**) selected area diffraction pattern taken from the grain marked with circle. Simulation of the solve for (**c**) AlNi and (**d**) Al_3_Ni_5_.

**Figure 11:**
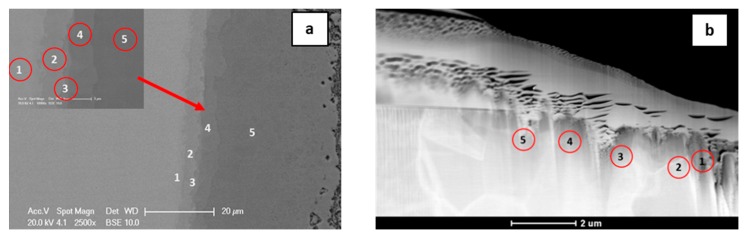
SEM (**a**) and TEM (**b**) microstructures of a Ni/Al/Ni interconnections (Ni substrate of B-type) after 3 h of reaction time at 720 °C with indicated EDS point analysis presented in Table 4. Numbers 1–5 denote particular intermetallic phases: 1-Ni solid solution, 2-AlNi_3_, 3-AlNi_Ni-rich_, 4-AlNi, 5-AlNi_Ni-deficient_.

The TEM investigation for sample annealed for 3 h at 720 °C indicates different results in comparison to the EBSD and EDS overlapping for sample after 20 h of annealing. The diffraction pattern from the area taken by the AlNi_Ni-rich_ phase (Figure 10), determined based on the chemical composition, did not unambigously confirmed its presence. Two possible phases were taken into consideration, namely AlNi and Al_3_Ni_5_. Important is that AlNi is always identified based on the same crystallographic data [31], however, the content of elements is different, so this phase is considered as AlNi without division into rich and deficient in nickel types. The degree of mismatch is very high for AlNi phase reaching 38% (Figure 10c). For the second considered phase Al_3_Ni_5_, the degree of mismatch is of only 9% (Figure 10d). This orthorombic phase is metastable below 700 °C, as the samples were cooled with furnace after the annealing process, it could be formed. However, due to the fact that growth of this phase takes place at 720 °C, in the manuscript it is noted as Ni-rich AlNi phase. Further TEM investigations would be of great benefit for the description and understanding of the phase evolution, especially for the early stage of the Ni-rich AlNi phase growth. As the phase thickness was below the analytical resolution in SEM (see Figure 11) such examination in TEM is of essential need. The results of TEM-EDS and SEM-EDS measurements are similar and were collected in Table 4, however, in case of the doubtful phase substantial difference is visible. From SEM-EDS it follows that the phase present in the joint is the Al_3_Ni_5_ but the TEM-EDS results point at the AlNi rich in nickel one. As mentioned above, this phase was extremely narrow, the thickness is on the border of the resolving power of the method. Examined areas for both methods are shown in Figure 11.

### 3.2. Growth Rate of the Intermetallic Phases

Determination of the thickness of the intermetallics (Table 5) allowed revealing their growth kinetics. For the short time of the reaction, the interconnection zones were broadening as it is assumed in the diffusion soldering process. Between 1 and 3 h of annealing only the subtle difference of the thickness of the whole joint was observed, nevertheless, different intermetallic phases in reaction zones appeared. Changes of phases composition points that the reaction after 1 h probably takes place in the solid state. After 5 h of annealing, due to isothermal solidification stage of DS, for both types of substrates, the interconnection zones shrink. As it was mentioned earlier, phases composition between 3 and 5 h stays the same, while the main difference is associated with the thickness of the interconnection zones for both types of substrates. For samples of A-type, the interconnection zones after 5 h of annealing is four times narrower and for B-type two times thinner than after 3 h of annealing. Comparing the samples after the same annealing conditions, for A-type to B-type Ni substrates, the following results are observed: after 1 h of annealing the thickness of the entire joint is comparable and widths of particular phases are similar. When time of annealing is extended to 3 h, the differences in diffusion process and in the overall appearance of the interconnection zones are not observed. After 5 h of annealing more visible differences appeared. First of all, the thickness of the joint, where substrate B-type was used, is twice broader than in Ni/Al/Ni reaction zone with A-type substrates. Additionally, in case of NiA/Al/NiA, the phase AlNi deficient in nickel is not observed. Thicknesses of the individual phases are similar, beside of the total width of AlNi phases (of every type), which in case of B-type nickel is broader, however, stoichiometric type of AlNi phases are comparable. This difference between thickness of whole joint after 5 h could be caused by the leakage of liquid solder during the experiment due to too high pressure applied. The comparison of the thickness of the particular layers of the intermetallic phases formed in Ni/Al/Ni interconnection in different time of reaction is collected in Table 5. Authors conducted experiment which allowed to eliminate the necessity of application of two separated systems: NiA/Al/NiA and NiB/Al/NiB. Numerous attempts prove that the localization of the Ni substrates (above or below the Al solder) does not affect the width and sequence of created phases. This approach resulted in simplifying the experimental procedure and allowed for producing of NiA/Al/NiB system (and conversely), which shortened experiment time. This procedure was used for shorter and longer annealing times. In early stages of diffusion soldering processes phase Al_3_Ni disappears fast—only after 30 min it is completely consumed and replaced by Al_3_Ni_2_ phase, which, in turn, after 3 h of annealing no longer exists. The longest times of annealing cause further phases broadening. Finally, after 72 h of annealing the interconnection zone contains only high nickel phases (50 at.% of Ni and higher). Three phases: AlNi_3_, AlNi, AlNi_Ni-rich_ for which the growth kinetic is calculated expand gradually with the annealing time.

**Figure 12:**
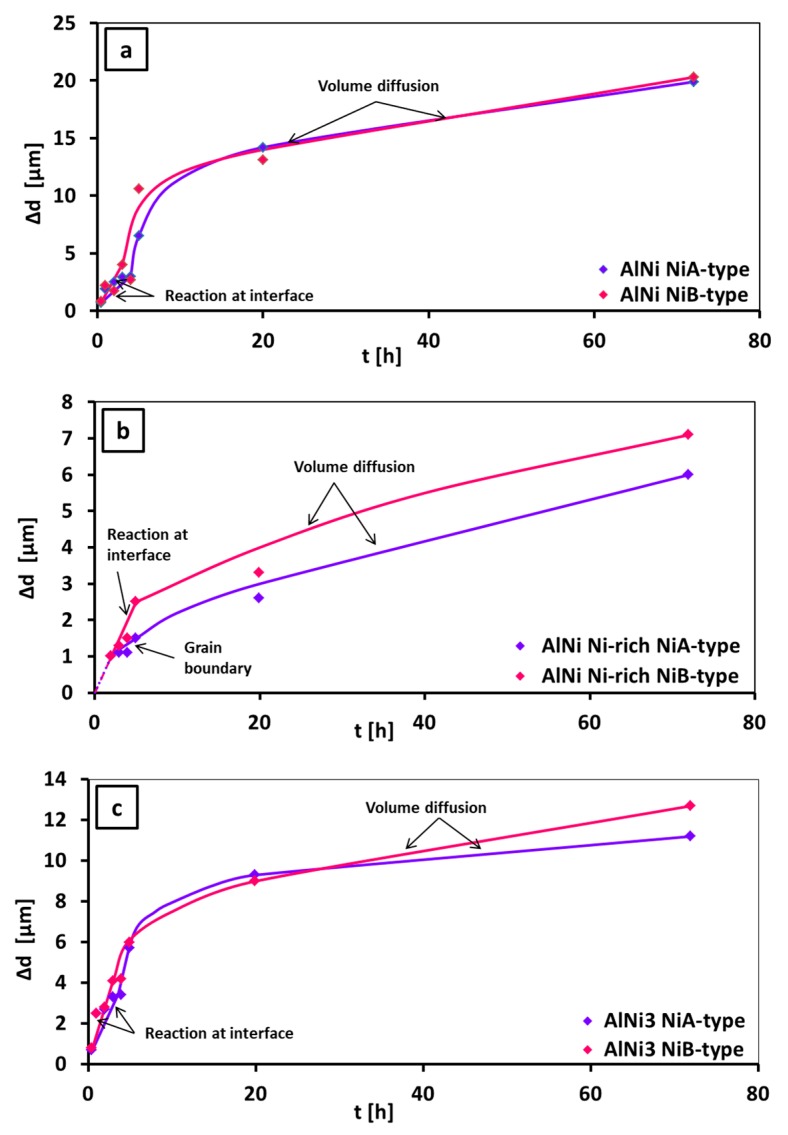
Time dependence of the layer width for stoichiometric AlNi (**a**), AlNi_Ni-rich_ (**b**) and AlNi_3_ (**c**) in Ni/Al/Ni joints.

Determination of the main mechanisms, which control the growth of the intermetallic phase is based on simple and useful formula (Equation 1), in details discussed in [32], giving the relation between the thickness of the intermetallics with the time of annealing.

(1) $$\Delta d=kt^{n}$$

where: Δd is a thickness of the intermetallic phase layer, k—the growth rate constant and t—time of annealing. To define the mechanism of the intermetallic phase growth, it is necessary to determine the value of n exponent. Depending on this value, the growth can be controlled either by the volume diffusion (n=0.5), or by the chemical reaction at the interfaces (n=1), grain boundary diffusion (n<0.5) and finally by mixed mechanism of growth (0.5<n<1). The growth rate constant k can be determined from two types of plot, namely Δd vs. $t^{\left(\frac{1}{2}\right)}$ or $\Delta d^{2}$ vs. t. First type of plot is better for the growth of the intermetallic phases, where the layer is formed at the initial period of growth and does not influence the course of further stabilized growth. Second type should be used, when the initial growth of the obtained layer affects the period of the parabolic growth [32]. Diffusion process may be controlled by the dislocation mechanism but only at lower temperature, while at the temperature close to the melting point of metals the amplitude of thermal vibrations of atoms is too high and dislocations as a structural effects disappear. Therefore, in such a case, the dislocations are not taken into account and the mechanisms of diffusion are either volume diffusion or diffusion by grain boundaries or reactive diffusion. The growth kinetics results are collected in Table 6 and shown in Figure 12.

The growth kinetics data for three phases formed in the solid state in Ni/Al/Ni interconnections was determined. Plots (Figure 12) Δd vs. t for AlNi stoichiometric (Figure 12a), AlNi_Ni-rich_ (Figure 12b) and AlNi_3_ phases (Figure 12c), showed that the growth of AlNi phases was controlled by different mechanism in comparison to AlNi_Ni-rich_ and AlNi_3_. The growth of AlNi phase involved two mechanisms: at first, the reaction at the interface took place and then it was replaced by the volume diffusion. The time exponent n for AlNi phase equals 0.67 for substrates of A-type and 0.65 for the B-type ones. The calculation showed that growth mechanism for AlNi rich in nickel and AlNi_3_ are similar and governed by the volume diffusion (n is 0.5±0.1). In the case of AlNi rich in nickel phase growth with the short incubation time occurred only for substrate of A-type. The AlNi_3_ phase grew due to the volume diffusion mechanism and no incubation time was observed in its growth. Finally, it was verified, that neither of these phases grew due to the grain boundary diffusion, therefore, the differences between the samples of A and B Ni substrates were not observed. In the study made by Lopez et al. [13] phases grew only by volume diffusion, while in present work the growth mechanism for the AlNi stoichiometric is found to be mixed (chemical reaction and volume diffusion). Lopez et al. [13] determined that the fastest growth occurred for stoichiometric AlNi and the slowest growth for Ni-rich AlNi one. They also calculated that the values of n factor equaled 0.5±0.1 for all three phases: AlNi, AlNi_Ni-rich_ and AlNi_3_. Therefore, the authors assumed that the growth of all these layers obeys a parabolic law—it is governed by the volume diffusion. What is interesting, they also noticed a transition period of AlNi_3_ growth for short time of annealing. In this study presented graphs in Figure 12 and Table 6 revealed new insight for the Al/Ni interaction. At present work the differences in behavior of phase-growth at short time of annealing and after longer time was noticed. Growth of AlNi rich in Ni phase is governed by volume diffusion but only after longer time of annealing. Focusing on the shorter time of process (2–5 h), the calculated n exponent shows that for substrates of A-type, the grain boundary mechanism dominates (n=0.37). On the other hand, considering of B-type substrate, data obtained at the beginning of DS suggesting the significant contribution of the reaction at interface (n=0.91). Similar behavior for AlNi_3_ phase for both types of substrates was observed, however in the range of 0.5–5 h time of annealing, only reaction at interface was evidenced as the governing mechanism of growth (for A-type n=0.73; for B-type n=0.76). In the case of AlNi stoichiometric, independently on time of annealing, two mechanisms of growth, as it was mentioned above - reaction at interface and volume diffusion occurred.

## 4. Conclusions

The diffusion soldering process was successfully applied to join nickel substrates using aluminum as the solder material at 720 °C for various periods of time. Nickel substrates, applied in the experiment, differed by size and shape of their grains in order to reveal any influence on the sequence of appearance or growth kinetics of particular intermetallic phases. It was demonstrated that at first the intermetallic phases rich in aluminum were formed (Al_3_Ni, Al_3_Ni_2_) and later replaced by the rich in nickel phases (AlNi, AlNi_3_). Three variants of AlNi phase were identified, all of them clearly visible in SEM-BSE: deficient in nickel (45 at. % of Ni), stoichiometric (50 at. % of Ni) and rich in nickel (60 at. % of Ni). While the Ni deficient AlNi phase formed separated layer of grains, electron backscattered diffraction map evidenced that the Ni-rich variant was located within the grains of already formed AlNi stoichiometric phase. Moreover, transmission electron microscopy examination revealed local presence of Al_3_Ni_5_ phase grains. Growth kinetics data showed that the fastest growing phase was the stoichiometric variant of AlNi phase, growing in a mixed mechanism: at first due to the chemical reaction and later by the volume diffusion. The most interesting results were obtained for two other phases: Ni-rich AlNi and AlNi_3_. As it was already showed in the literature, the general growth can be attributed to the volume diffusion, however, at early stages significant differences were noticed in this study. In the case of AlNi_3_ phase, the first stage of growth was due to the chemical reaction and later it slowed down (volume diffusion). For the Ni-rich AlNi phase in the later stage of growth the volume diffusion dominated. However, the first stage of phase growth varied in relation to the applied nickel substrate. In the interconnections, for which the surface of contact with aluminum was composed of the elongated grains, the grain boundary diffusion mechanism occurred, while for the Ni with small, equiaxed grains, the chemical reaction governed the growth of the phase. This work demonstrates that different mechanisms may contribute in the growth of the phases, especially in the beginning of the process and one should be aware of this. The substrate microstructure may have the influence on the intermetallics’ growth kinetics and resulting from it differences in the integral diffusion coefficients.

## Figures and Tables

**Table 1:** The experimental assemblies and conditions. NiA denotes the substrate cut to the parallel direction to the elongation rod, while the NiB in the perpendicular direction.

Conditions:			Temperature 720 °C, Vacuum, Slight Mechanical Pressure, Heating 18 °C/min Cooling with Furnace
No.	Sample Type		Time of Annealing [h]
1.	NiA/Al/NiB		0.25
2.	NiA/Al/NiB		0.50
3.	NiA/Al/NiA	NiB/Al/NiB	1
4.	NiA/Al/NiB		2
5.	NiA/Al/NiA	NiB/Al/NiB	3
6.	NiA/Al/NiB		4
7.	NiA/Al/NiA	NiB/Al/NiB	5
8.	NiA/Al/NiB		20
9.	NiA/Al/NiB		72

**Table 2:** Average thickness of the whole joint and particular intermetallic phases—both sides of the joint.

Phase	Present Work	Tumminello et al. [14]
	Thickness [μm]	
Whole interconnection zone	280	140
Eutectics Al + Al_3_Ni	86	56
Al_3_Ni	15	9
Al_3_Ni_2_	82	33

**Table 3:** Chemical composition of the intermetallic phases in Ni/Al/Ni joints after annealing for different periods of time.

Phase	The Range of Aluminum Content (at. %) in Interconnection	Average Content of Aluminum in the Interconnection (at. %)
Ni solid	5.5–12.1 ± 0.2–0.5	8.9 ± 0.2
AlNi_3_	21.6–30.5 ± 0.4–0.6	26.1 ± 0.5
AlNi_Ni-rich_	33.4–42.0 ± 0.7–0.8	39.1 ± 0.8
AlNi	47.1–53.5 ± 0.9–1.1	50.6 ± 1.0
AlNi_Ni-deficient_	53.7–57.8 ± 1.1–1.2	55.9 ± 1.1
Al_3_Ni_2_	58.2–60.7 ± 1.2	59.6 ± 1.2
Al_3_Ni	75.5–76.3 ± 1.5	76.0 ± 1.5

**Table 4:** Comparison of the chemical compositions obtained by SEM and TEM.

No.	Method	SEM		TEM	
	Phase	Content at. %			
		Ni	Al	Ni	Al
1.	Ni solid solution	94.4 ± 1.9	5.6 ± 1.9	91.9 ± 1.8	8.1 ± 1.8
2.	AlNi_3_	78.4 ± 1.6	21.6 ± 1.6	78.4 ± 1.6	21.6 ± 1.6
3.	AlNi_Ni-rich_	66.6 ± 1.3	33.4 ± 1.3	60.8 ± 1.2	39.2 ± 1.2
4.	AlNi	50.1 ± 1.0	49.9 ± 1.0	51.9 ± 1.0	48.1 ± 1.0
5.	AlNi_Ni-deficient_	45.6 ± 1.0	54.4 ± 1.0	45.8 ± 0.9	54.2 ± 0.9

**Table 5:** The thickness of the particular layers of the intermetallic phases formed in Ni/Al/Ni interconnection in different time of reaction at 720 °C.

Time [h]	Ni-Type	Layer Thickness, Δd [μm]					
		Al_3_Ni	Al_3_Ni_2_	AlNi_Ni-deficient_	AlNi	AlNi_Nirich_	AlNi_3_
0.25	A	16.9	87.8	-	-	-	-
	B	12.9	76.5	-	-	-	-
0.50	A	-	94.2	-	0.7	-	0.7
	B	-	96.3	-	0.8	-	0.8
1	A	-	40.2	-	1.9	-	2.5
	B	-	50.4	-	2.2	-	2.5
2	A	-	108.7	-	2.5	1.0	2.7
	B	-	103.7	-	1.7	1.0	2.8
3	A	-	-	48.1	2.9	1.1	3.3
	B	-	-	38.1	4	1.3	4.1
4	A	-	-	77.6	3	1.1	3.4
	B	-	-	45.5	2.7	1.5	4.2
5	A	-	-	-	6.5	1.5	5.7
	B	-	-	7.2	10.6	2.5	6
20	A	-	-	7.5	14.2	2.6	9.3
	B	-	-	10.5	13.1	3.3	9
72	A	-	-	-	19.9	6.0	11.2
	B	-	-	-	20.3	7.1	12.7

**Table 6:** The growth kinetics of AlNi, AlNi_Ni-rich_ and AlNi_3_.

Ni A-Type	n	k	Mechanism	Ni B-Type	n	k	Mechanism
AlNi	0.67	$1.51\frac{\mu m^{0.67}}{h}$	mixed: volume diffusion and chemical reaction at interface	AlNi	0.65	$1.65\frac{\mu m^{0.65}}{h}$	mixed: volume diffusion and chemical reaction at interface
AlNi_Ni-rich_	0.51	$1.59\frac{\mu m^{0.51}}{h}$	volume diffusion	AlNi_Ni-rich_	0.52	$1.28\frac{\mu m^{0.52}}{h}$	volume diffusion
AlNi_3_	0.51	$1.77\frac{\mu m^{0.51}}{h}$	volume diffusion	AlNi_3_	0.50	$1.96\frac{\mu m^{0.50}}{h}$	volume diffusion
